# Supplementary material for: A fault-intrusion-tolerant system and deadline-aware algorithm for scheduling scientific workflow in the cloud
Source: PeerJ Comput Sci. 2021 Nov 2;7:e747. doi: 10.7717/peerj-cs.747 (PMC8576554; doi:10.7717/peerj-cs.747)
Supplement: Supplemental Information 6 [file peerj-cs-07-747-s006.zip › Myproject1.1/docs/org/workflowsim/utils/class-use/ReplicaCatalog.FileSystem.html]

Uses of Class org.workflowsim.utils.ReplicaCatalog.FileSystem


JavaScript is disabled on your browser.


- Overview
- Package
- Class
- Use
- Tree
- Deprecated
- Index
- Help

- Prev
- Next

- Frames
- No Frames

- All Classes

## Uses of Class org.workflowsim.utils.ReplicaCatalog.FileSystem

- Packages that use ReplicaCatalog.FileSystem

  | Package | Description |
  | --- | --- |
  |  |  |
  | --- | --- |
  | org.workflowsim.utils |  |
- - ### Uses of ReplicaCatalog.FileSystem in org.workflowsim.utils

    Methods in org.workflowsim.utils that return ReplicaCatalog.FileSystem

    | Modifier and Type | Method and Description |
    | --- | --- |
    |  |  |
    | --- | --- |
    | `static ReplicaCatalog.FileSystem` | ReplicaCatalog.`getFileSystem()` Gets the file system |
    | `static ReplicaCatalog.FileSystem` | ReplicaCatalog.FileSystem.`valueOf(java.lang.String name)` Returns the enum constant of this type with the specified name. |
    | `static ReplicaCatalog.FileSystem[]` | ReplicaCatalog.FileSystem.`values()` Returns an array containing the constants of this enum type, in the order they are declared. |

    Methods in org.workflowsim.utils with parameters of type ReplicaCatalog.FileSystem

    | Modifier and Type | Method and Description |
    | --- | --- |
    |  |  |
    | --- | --- |
    | `static void` | ReplicaCatalog.`init(ReplicaCatalog.FileSystem fs)` Initialize a ReplicaCatalog |

- Overview
- Package
- Class
- Use
- Tree
- Deprecated
- Index
- Help

- Prev
- Next

- Frames
- No Frames

- All Classes
